# Supplementary material for: Variations in the constituent year effect in Junior World Championships in alpine skiing: A window into relative development effects?
Source: PLoS One. 2020 Apr 30;15(4):e0231384. doi: 10.1371/journal.pone.0231384 (PMC7192383; doi:10.1371/journal.pone.0231384)
Supplement: Attachment — Submitted filename: Reviewer 2 review.pdf [file pone.0231384.s001.pdf]

Manuscript Number: PONE-D-19-22259
Article Type: Research Article
Full Title: Variations in the Constituent Year Effect in Junior World Championships in Alpine Skiing
Corresponding Author: Øyvind Bjerke Norges teknisk-naturvitenskapelige universitet
Trondheim, NORWAY

Summary

The majority of research across all sport contexts regarding the overrepresentation of athletes born earlier in the cohort and the underrepresentation of athletes born late in the cohort, known as the relative age effect (RAE), have focused on 12-month competition seasons (within one-year effect) (Cobley, Baker, Wattie & McKenna, 2009; Korgaokar, Farley, Fuller, & Caputo, 2018). In contrast, fewer studies have been published on the constituent year effect (CYE), which describes the selection rate among early-born and late-born athletes across multiyear age bands (e.g. 16-19 years of age). Consequently, more research is needed to address the CYE across sports that use multiple age bands. The current study contributes to the literature on CYE among junior athletes and provides necessary insight into the potential selection bias.

The introduction and the abstract have clearly identified the need for more research on the CYE among elite level athletes. The methodology is consistent with other research in the same area and addresses the main questions in the study. The results are clear and logically presented and all figures and tables appropriately labeled. The conclusion addresses the main question(s) in the study; however, there is no mention of potential mechanisms to attenuate or eliminate RAE and CYE among elite-level junior Alpine skiers (Wattie, Schorer, & Baker, 2015).

In closing, I recommend this paper be approved for publication pending additional reviewer recommendations and comments.

Major Issues

There are no major flaws in this research and I recommend this study be approved for publication.

Minor Issues

Address potential mechanisms or studies that have been implemented to reduce RAE and CYE among junior elite level athletes (Wattie, Schorer, & Baker, 2015).

Several of the references are not in correct format. Please see the following reference line numbers that need editing: 327, 361, 363, 369, 374, 383, 385, 404.
Other Comments

The title is appropriate and addresses the key questions in this study.

References

Cobley, S., Baker, J., Wattie, N., & McKenna, J. (2009). Annual age-grouping and athlete development. Sports Medicine, 39(3), 235-256.

Korgaokar, A. D., Farley, R. S., Fuller, D. K., & Caputo, J. L. (2018). Relative age effect among elite youth female soccer players across the United States. Sport Mont, 16(3), 37-41.

Wattie, N., Schorer, J., & Baker, J. (2015). The relative age effect in sport: A developmental systems model. Sports Medicine, 45(1), 83-94.
