# Supplementary material for: Variations in the constituent year effect in Junior World Championships in alpine skiing: A window into relative development effects?
Source: PLoS One. 2020 Apr 30;15(4):e0231384. doi: 10.1371/journal.pone.0231384 (PMC7192383; doi:10.1371/journal.pone.0231384)
Supplement: Attachment — Submitted filename: renamed_80aa3.docx [file pone.0231384.s002.docx]

# Point-by-point response to comments:

Reviewer #1:

Comment #1. The authors could explore recent research on relative age effect.
Response #1: We have already included a substantial amount of previous papers on research on relative age effect. However, in the revised manuscript, we have included some recent studies that have been published during the review process.

Comment #2. In addition, Wattie et al. (2015) presents an interesting theoretical model that can help support the study hypotheses. (DOI 10.1007/s40279-014-0248-9)
Response #2: We thank the reviewer for suggesting this highly relevant paper and we have attempted to incorporate that perspective into the revised manuscript.

Comment #3. Please identify what your classifications were for interpreting effect size.
Response #3: As requested, we have included classifications for interpreting effect sizes based upon Cohen (2013).

Comment #4. I really think the pictures are of poor quality. Please use specific software to build graphics and improve overall quality.
Response #4: We have redone the figures with improved quality, as requested.

Comment #5. The discussion addresses the main points of the data and interpretation. I simply question the practical implications of these findings. I think that the authors could take a small approach about this.
Response #5: We have included in the discussion a short paragraph discussing practical implications, focusing on possible suggestions for reducing the impact of RAE and CYE. Please see line 310-316 in revised manuscript.

Comment #6. Please, make sure you follow the formatting guidelines for references. Some misconceptions were seen in the following references: 4, 18, 21, 23, 28, 35.

Response #6: We have checked the reference list and adjusted in accordance with formatting guidelines.

Reviewer #2:

Comment #7. Address potential mechanisms or studies that have been implemented to reduce RAE and CYE among junior elite level athletes (Wattie, Schorer, & Baker, 2015).

Response #7: We have included in the discussion a short paragraph where these mechanisms are adressed. Please see line 310-316 in revised manuscript.

Comment #8. Several of the references are not in correct format. Please see the following reference line numbers that need editing: 327, 361, 363, 369, 374, 383, 385, 404.

Response #8: We have checked the reference list and adjusted in accordance with formatting guidelines.
